# Genome-Wide Hierarchy of Replication Origin Usage in Saccharomyces cerevisiae

**DOI:** 10.1371/journal.pgen.0020141

**Published:** 2006-09-08

**Authors:** Justin J Donato, Shau Chee C Chung, Bik K Tye

**Affiliations:** Department of Molecular Biology and Genetics, Cornell University, Ithaca, New York, United States of America; National Institute of Diabetes and Digestive and Kidney Diseases, United States of America

## Abstract

Replication origins in a genome are inherently different in their base sequence and in their response to temporal and cell cycle regulation signals for DNA replication. To investigate the chromosomal determinants that influence the efficiency of initiation of DNA replication genome-wide, we made use of a reverse strategy originally used for the isolation of replication initiation mutants in *Saccharomyces cerevisiae.* In yeast, replication origins isolated from chromosomes support the autonomous replication of plasmids. These replication origins, whether in the context of a chromosome or a plasmid, will initiate efficiently in wild-type cells but show a dramatically contrasted efficiency of activation in mutants defective in the early steps of replication initiation. Serial passages of a genomic library of autonomously replicating sequences (ARSs) in such a mutant allowed us to select for constitutively active ARSs. We found a hierarchy of preferential initiation of ARSs that correlates with local transcription patterns. This preferential usage is enhanced in mutants defective in the assembly of the prereplication complex (pre-RC) but not in mutants defective in the activation of the pre-RC. Our findings are consistent with an interference of local transcription with the assembly of the pre-RC at a majority of replication origins.

## Synopsis

The length of S phase regulated by the rate of DNA synthesis varies dramatically during the development of metazoans. Key to this regulation is the number of replication origins utilized in different developmental stages. A fundamental question is whether there is a hierarchy in the usage of replication origins under different conditions and if so, what are the determinants for preferential usage. In *Saccharomyces cerevisiae,* replication origins isolated in DNA fragments are known as autonomously replicating sequences (ARSs). To gain insight into the determinants that regulate replication origin usage, genomic ARSs that are preferentially used under adverse conditions for replication initiation were identified. One of the determinants appears to be the local transcription pattern. Transcriptional activity directed towards an ARS correlates with reduced efficiency of replication initiation of that ARS. This transcriptional interference appears to be targeted at the assembly of the prereplication complex. These results are consistent with the deregulated initiation patterns observed in early developing *Xenopus* embryos that are devoid of transcription. Other yet-to-be-identified factors are also important in determining the efficiency of replication origin usage.

## Introduction

The rate of DNA replication and the rate of cell proliferation are dependent on the frequency of initiation events or density of replication origins utilized. This correlation has been observed in prokaryotes [1] and during development in metazoans [2,3]. Little is known about the regulatory mechanisms for selective origin usage in response to environmental or developmental cues in metazoans. In *Saccharomy*ces *cerevisiae,* a direct correlation between the length of S phase and the size of replicons has been demonstrated [4]. Furthermore, not all replication origins are initiated at an equivalent efficiency [5]. This differential usage of replication origins is responsive to conditions unfavorable to replication initiation [6–8].

All origins of replication in S. cerevisiae contain an 11-bp AT-rich consensus element, the autonomously replicating sequence (ARS) consensus sequence; however, the presence of this element alone is not sufficient to define a replication origin [9]. A number of recent studies have attempted to identify the chromosomal locations of all of the potential replication origins in the S. cerevisiae genome (reviewed in [10]). One approach used density transfer or copy number increase to identify newly replicated regions as initiation sites at a genomic scale [11,12]. Another approach used chromatin immunoprecipitation experiments to identify the genomic locations of known replication initiation factors [13]. A third approach used computational analysis to predict locations of replication origins based upon the sequences of a set of known ARSs [9]. Each of these methods has its limitations and only one relies on functional evidence. The first approach has a limitation of resolution of greater than 10 kb. The second is likely to represent an overestimation since many replication initiation proteins have functions other than replication initiation [14,15]. The third is limited by the choice of ARSs used in the trainer set of the computation analysis. However, the combined dataset from all of these approaches is likely to represent an almost complete array of the genomic replication origins.

In addition to the ARS consensus sequence, every origin contains a set of flanking domains that enables it to serve as a site for DNA replication initiation [16,17]. While the exact mechanism of enhancement remains unknown, some well-characterized elements have been shown to be transcription factor binding sites, while others are regions of DNA unwinding [18–21]. It is unclear how large a contribution ARS-flanking regions make to replication initiation as well as how far away they lie from the ARS consensus sequence. It appears that the flanking regions of certain origins have a capacity to overcome conditions compromised for replication initiation [8]. Many replication origins show reduced initiation efficiency in mutants defective in the DNA replication initiation machinery, while others function normally [22]. This difference is most likely due to the nature of the enhancer elements present at each individual origin as well as the chromosomal context surrounding the origin.

Eukaryotic organisms have evolved elaborate mechanisms to achieve the levels of regulation needed to ensure the fidelity of their replicated genomes. Using the budding yeast as a model system, one of the original genetic screens for mutants exhibiting replication defects identified a set of minichromosome maintenance (*mcm*) mutants [6]. The mutants are unable to efficiently initiate replication of plasmids containing certain replication origins, while allowing some replication origins to initiate DNA synthesis as efficiently as they do in wild-type [7,23,24]. Subsequent work has shown that this differential initiation defect applies to ARSs on plasmids as well as their corresponding chromosomal locations [8,25]. These mutants affect different steps in the initiation of DNA synthesis, and their effects are manifested to different levels of severity at different origins. For example, *mcm2–1* [7]*, mcm3–1* [26], and *mcm5–1* [27] are mutants of genes that encode subunits of the hexameric MCM helicase [22]. They are components of the prereplication complex (pre-RC) that is recruited to replication origins during late M or early G_1_ phase [25]. Another mutant is *mcm1–1. MCM1* encodes a transcription factor that binds the flanking regions of replication origins to facilitate efficient initiation [8,20]. Interestingly, all of these *mcm* mutants appear to have the same preference for ARS usage.

In this study, *mcm1–1* cells were used as a tool to highlight distinguishing features between ARSs that enable their differential usage under suboptimal initiation conditions. A genomic library was constructed in which the insert sizes were large enough to contain intact ARSs along with their flanking sequences. This library was then propagated in *mcm1–1* cells to select for plasmids containing ARSs that behave as they do in wild-type cells. Until this point, only two such initiation “robust” ARSs, ARS121 and the telomeric ARS120, had been identified [8]. The main goal of this screen was to identify additional robust ARSs at a genomic scale so as to determine the common elements that allow these ARSs to initiate under conditions when DNA replication is compromised. Our results show that there is a correlation between replication efficiency at a given ARS and its local transcription pattern. Preferential usage of ARSs is most evident in mutants defective in the assembly step of the pre-RC but not in the activation step of the pre-RC. These results suggest that replication initiation is most sensitive to transcriptional interference during the assembly of the pre-RC.

## Results

### ARSs that Initiate Efficiently Are Selectively Enriched from a Genomic DNA Library in *mcm1–1* Cells

**Figure 1:**
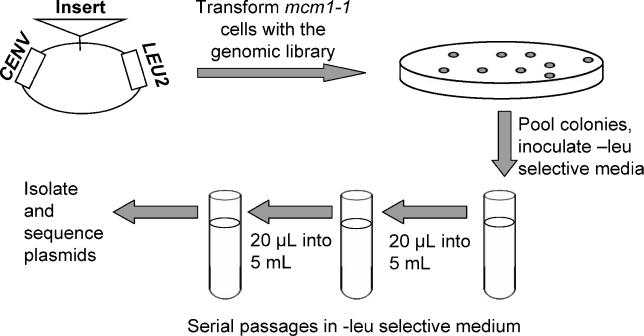
Serial Passages of a Plasmid Library of Genomic DNA in *mcm1–1* Cells to Enrich for Stable Plasmids Containing A-Type ARSs

Genomic ARSs can be divided into two categories, A-type and B-type. A-type ARSs replicate efficiently in the *mcm1–1* mutant, while B-type ARSs replicate poorly [6]. To determine the characteristics of A-type ARSs that facilitate their efficient initiation in the *mcm1–1* mutant background, it was necessary to collect a larger sample for analysis. A genetic screen to identify these origins is outlined in Figure 1. The rationale is that cells containing plasmids that replicate efficiently should segregate a copy of that plasmid to their daughter cells at a higher rate than a poorly replicating plasmid. After selecting for cells containing plasmids for ~35 generations, predominantly stably replicating or A-type plasmids would be expected to be present in the final culture.

**Figure 2:**
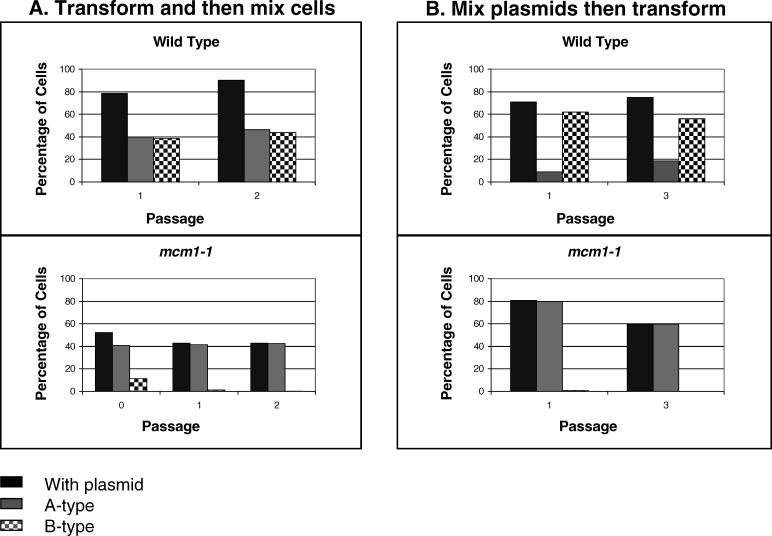
YCp121 Is More Stable than YCp1 in *mcm1–1* Cells Cells (wild-type or mutant) containing YCp121 or YCp1 were mixed (A). YCp121 and YCp1 were mixed and transformed into wild-type or *mcm1–1* cells (B). Colonies on selective medium were pooled and used to inoculate a culture in selective media. The culture was grown to saturation (one passage) and used to inoculate a fresh culture of selective media. This process was repeated two or three times. The relative abundance of YCp1 containing ARS1 and YCp121 containing ARS121 was measured at the end of a passage.

As a proof of principle, two approaches were taken. First, we transformed wild-type and mutant cells with YCp121 or YCp1. YCp121 is a stably replicating plasmid that contains ARS121, an A-type ARS, as well as the *LEU2* gene [6]. YCp1 is an unstable plasmid that contains ARS1, a B-type ARS, as well as both *LEU2* and *URA3* [6]. We mixed two similar-sized colonies, one containing YCp121 and the other containing YCp1 that had been grown on medium lacking leucine. The mixture (0 passage) was used to inoculate a fresh culture of selective media lacking leucine to generate overnight inoculants (first passage). This process was repeated and the cultures underwent two passages. After each passage, an aliquot was taken and plated on rich media, on media lacking leucine, and on media lacking uracil. By counting the resulting colonies the percentage of cells in each culture that contained each plasmid was calculated. As shown in Figure 2A, in the wild-type cells, neither plasmid affords the cells a clear growth advantage on selective media. In contrast, in *mcm1–1* cells, plasmids carrying YCp121 greatly outnumber plasmids carrying YCp1 even in the initial mixture (0 passage), suggesting that YCp1 was unstable in the initial colony. In a second approach, we constructed a pool of plasmids containing both YCp121 and YCp1 in a ratio of about 1:6 (Figure 2B). The mixture was used to transform both wild-type and *mcm1–1* cells. In both cases, the transformants were pooled and used to inoculate a fresh culture of selective media to generate overnight inoculants. This process was repeated twice, and the cultures underwent three passages. The percentages of cells carrying each of the plasmids in each of the strains were scored as described above. As observed in the first approach, there is a clear growth advantage for cells carrying YCp121 in the *mcm1–1* mutant, indicating that A-type ARSs are enriched under this selection scheme.

### A Genomic Library Was Constructed and Screened for Stable Plasmids in *mcm1–1* Cells

To search for A-type ARSs, a plasmid library was cloned into a vector backbone containing a centromere and a marker gene, as described in Materials and Methods (Figure 1). The genomic library was used to transform *mcm1–1* cells. Plasmids containing functional ARSs gave rise to colonies that propagated robustly in selective media. Transformants were allowed to grow for 2–3 d before being pooled to inoculate a single culture of selective media. This culture was then used to inoculate fresh selective media according to the scheme in Figure 1. This enrichment process of serial passages should selectively propagate plasmids containing A-type ARSs to the exclusion of plasmids containing B-type ARSs. To verify this premise, plasmids were isolated from the yeast culture and sequenced. In a control study aimed at identifying both B- and A-type ARSs, the library was used to transform wild-type yeast. Transformants were pooled and grown in a single culture, and plasmids were extracted from the culture without further enrichment. The resulting plasmids in this control study were sequenced to identify the ARS present on each insert.

Table S1 contains a list of all of the plasmids isolated from both wild-type and *mcm1–1* cells. The chromosomal coordinates of the ends of the inserts, the ARS, and plasmid loss rates are also listed. Table 1 shows relevant statistics on the plasmids obtained from wild-type and mutant cells combined. More than 470 plasmids have been isolated and sequenced, giving rise to 210 unique inserts representing 142 ARSs or functional origins of replication. While most (189) plasmids contain only one ARS, some (21) were found to have more than one ARS. Inserts containing a single ARS ranged in size from 134 bp to 20.65 kb, with an average size of 5.57 kb, suggesting that the majority of the ARS inserts in this library contain sufficient flanking sequences that contribute to the initiation efficiency of their corresponding chromosomal replication origins.

**Table 1:**
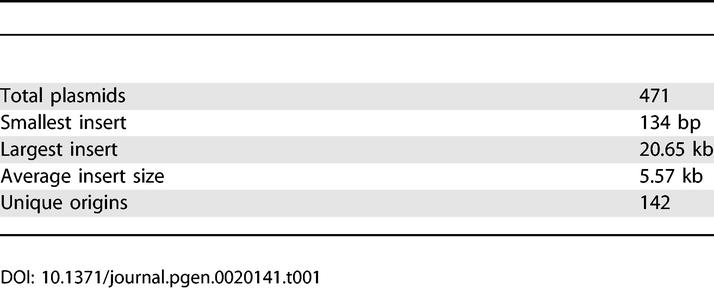
Relevant Statistics on Plasmids Isolated from Both Wild-Type and *mcm1–1* Cells

While there is significant redundancy between the lists of potential origins reported by three independent studies [9,11,13], we were able to isolate three novel ARSs. The first of these ARSs was named ARS207.5. It was isolated on plasmid #246 from the wild-type strain. This plasmid contains an insert corresponding to the region of Chromosome 2 between 175780 and 182448. The second of these ARSs was named ARS1114.5, which was isolated more than once from both the mutant and wild-type strain. The ARS1114.5 insert on plasmid #38 corresponds to the region of Chromosome 11 between 454458 and 459202. The last of these ARSs was named ARS1618.5. It was isolated on plasmid #187 from the wild-type strain. This plasmid contains an insert from Chromosome 16 between 414034 and 416074.

### Plasmids Containing More Than One ARS Have Increased Efficiency of Propagation

Once a plasmid was isolated and identified, its stability in *mcm1–1* cells was tested using the mcm assay. A plasmid was deemed to be stable (A-type) if its loss rate per generation was at or below 0.15 (see below). Sixteen plasmids were isolated with inserts that contain two previously identified ARSs (Table 2). In most cases tested, plasmids that contain two ARSs are stably propagated in mutant cells. These plasmids were therefore eliminated from further analysis. Fourteen of the 16 plasmids listed in Table 2 fall into this category. The exceptions are plasmids #169 and #166. These plasmids showed a loss rate that is higher than would be expected for plasmids with two functional ARSs. Each of these plasmids contains two potential replication origins based on a genome-wide location study of known replication initiation factors [13]. However, since genome-wide location study does not provide functional information of these potential replication origins, these designations can only be viewed as tentative and represent an overestimation of the number of functional origins in the genome. Indeed, each of two other studies reported only a single origin of replication for each of the inserts on these two plasmids [9,11]. Therefore, it is likely that these two plasmids actually contain only a single B-type ARS, accounting for the elevated plasmid loss rate.

**Table 2:**
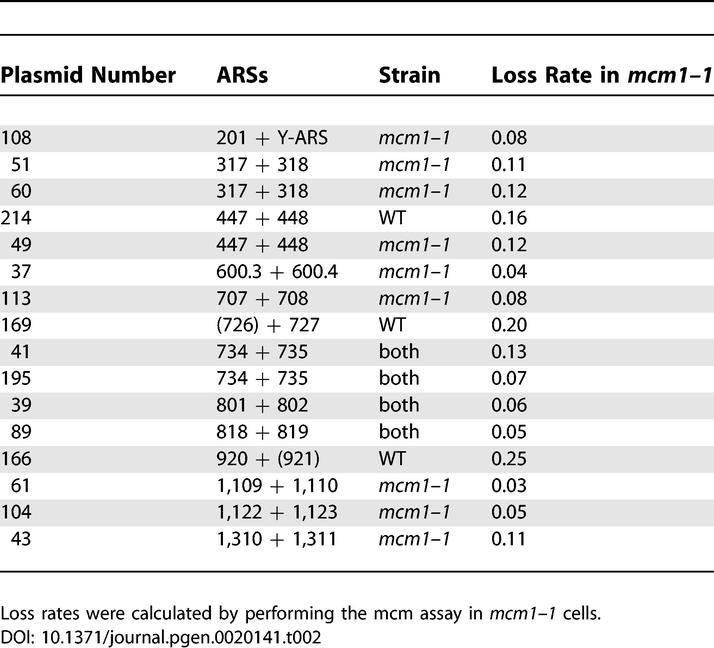
Plasmids Containing Two Potential ARSs and Their Corresponding Loss Rates per Cell Division

### Insert Size Influences Plasmid Stability

**Figure 3:**
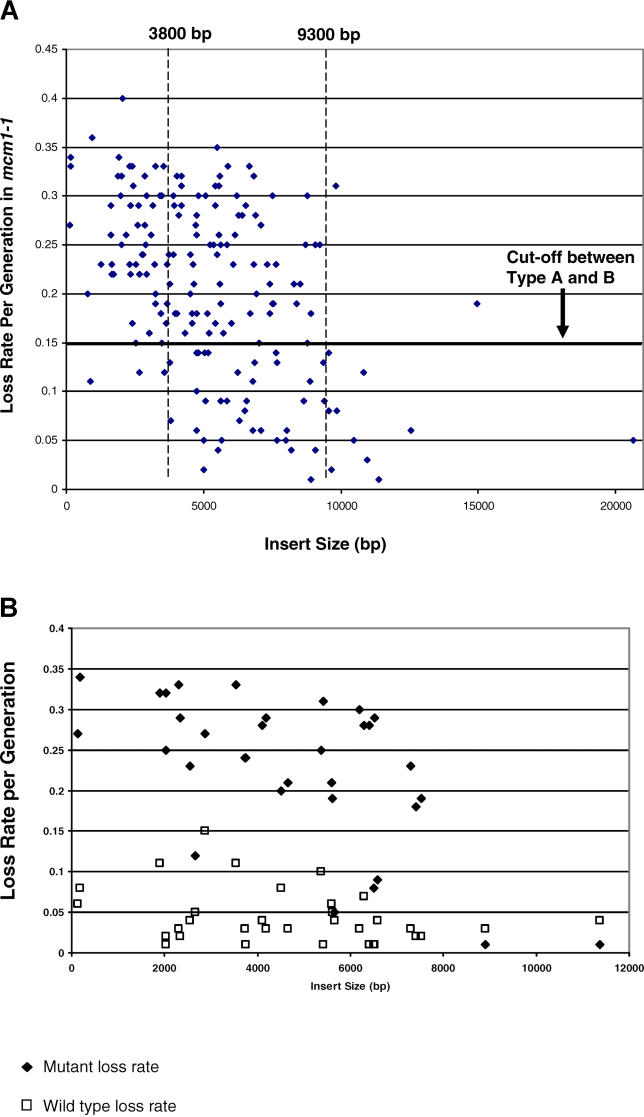
Characterization of A-Type and B-Type ARSs (A) Effect of insert size on plasmid stability. Loss rates of plasmids per cell division in *mcm1–1* cells plotted against insert size. Only inserts between 3,800 and 9,300 bp were analyzed further. (B) Stability of plasmids was measured in mutant (black diamonds) and wild-type (white squares) cells.

To investigate whether plasmid size influences stability, plasmid stability in the *mcm1–1* mutant versus insert size was plotted for all of the plasmids that were isolated and deemed to contain a single ARS (Figure 3A). A plasmid (#52) containing ARS121 on a 7,079-bp insert was isolated as a stable plasmid, exhibiting a loss rate of 0.06 in *mcm1–1* cells; another plasmid (#27) containing ARS121 on a smaller insert (2,332 bp) was isolated from the wild-type culture and had a much higher loss rate of 0.29 in *mcm1–1* cells. As shown in Figure 3A, there is a correlation between insert size and plasmid stability. In 91% of the stable plasmids isolated, the insert size is greater than 3,800 bp. On the other hand, in 90% of the plasmids with inserts greater than 9,300 bp, the plasmid was found to be stable in the mutant background regardless of the ARS present on the insert. Despite the size discrimination observed in large and small plasmids, there is no correlation between insert size and plasmid stability for plasmids whose inserts fall between 3,800 and 9,300 bp. For these reasons, only plasmids from within this range were analyzed further to minimize bias based solely on insert size.

Another consideration for the influence of large size is that because there are origins in the genome that have yet to be identified, it is possible that these larger inserts contain multiple yet-to-be-identified functional origins. This is unlikely to be true for all of the large inserts identified. Of the 20 inserts that were larger than 9,300 bp, 15 contain only one known origin of replication. All of these plasmids are verified to be stable plasmids by the mcm assay. This study has found three new ARSs out of 142 ARSs identified, suggesting that approximately 98% of the origins in the genome have been reported. Therefore, it is unlikely that all 15 of the large inserts also contain an unknown ARS.

### A-Type versus B-Type ARSs

Classification of A-type versus B-type ARSs was determined based on a plot of the loss rates of each plasmid in wild-type and mutant cells (Figure 3B). No plasmid has a loss rate higher than 0.15/cell division in wild-type cells. Plasmids containing A-type ARSs show similar loss rates in both wild-type and mutant cells, whereas those containing B-type ARSs show a clear difference. Based on this analysis, ARSs of plasmids that have a loss rate of less than 0.15 in mutant cells are classified as A-type ARSs, and those that have a loss rate of greater than 0.15 as B-type. In all, the enrichment screen in the *mcm1–1* mutant accounts for 88% of the A-type ARSs identified (Table 3), whereas 93% of the B-type ARSs were isolated from wild-type cells (Table 4).

**Table 3:**
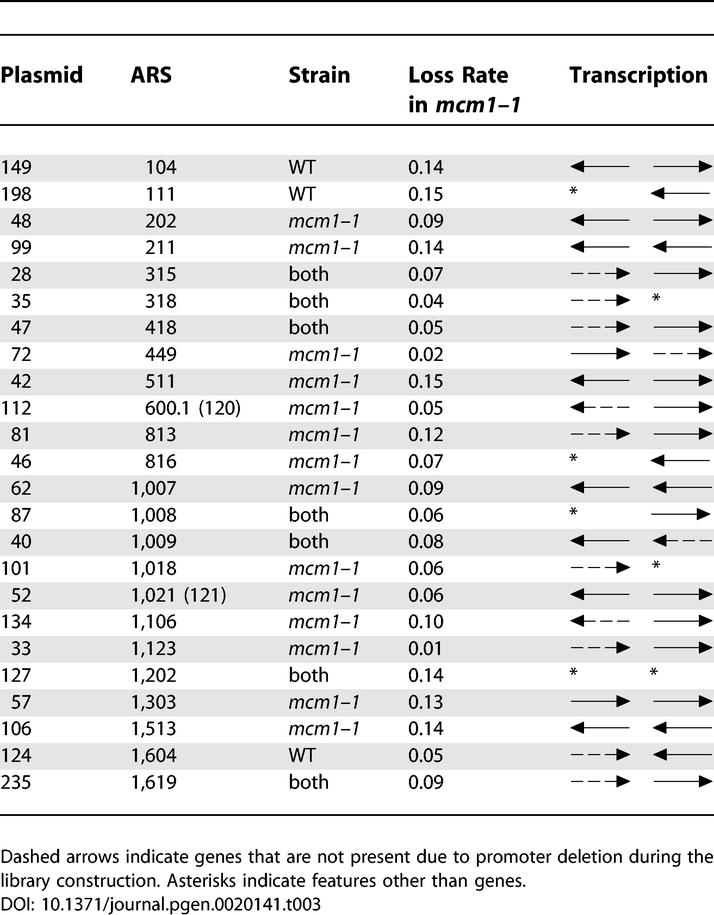
Characterization of Stable Plasmids Containing A-Type ARSs

**Table 4:**
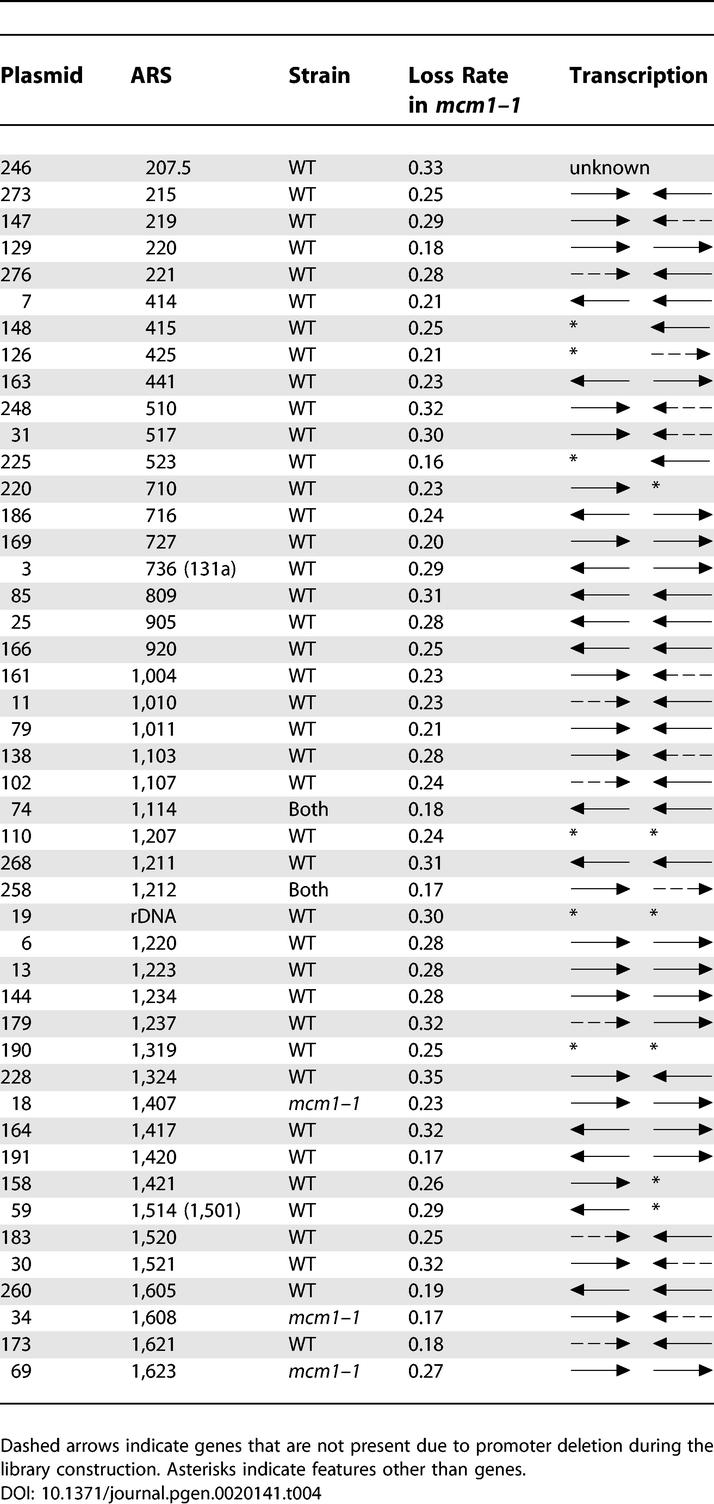
Characterization of Unstable Plasmids Containing B-type ARSs

To estimate the proportion of A-type versus B-type ARSs in the genome, we divided the number of A-type ARSs isolated from wild-type cells by the total number of ARSs isolated from wild-type cells. We found that 11/56, or 20% of the ARSs isolated, are A-type ARSs that initiate efficiently in the mutant background. Until this screen was performed, the only known A-type ARSs were ARS121 and ARS120. Both of these ARSs were isolated as stable plasmids (plasmids #52 and #112) from the *mcm1–1* mutant, thus confirming the validity of our screen. By combining the total number of A-type ARSs isolated from both mutant and wild-type strains, we have increased the number of A-type ARSs from 2 to 24 in this study (Table 3). Twenty-one of these A-type ARSs were isolated from plasmid enrichment in the *mcm1–1* strain. This sample size approaches that required for a more complete analysis of the characteristics that contribute to the activation efficiency of ARSs in general.

### Orientation of Transcription Units Correlates with ARS Activity

To investigate the influence of local transcriptional activity on ARS function [28,29], the remaining bona fide A-type and B-type ARSs were classified according to the orientation of adjacent genes. If a gene on either side was oriented such that the transcription bubble would move towards the ARS, then the ARS was considered to be in an active transcription zone. On the other hand, if no transcription bubble was present moving towards the ARS, then the ARS was classified as being in a nontranscribed region. The local transcription profile for each of the A-type “stable” ARSs is depicted in the last column of Table 3. It was found that 16/24, or 67% of the stable plasmids listed, are in regions devoid of approaching transcription bubbles. Some of these regions normally would be transcribed in their native chromosomal loci but were rendered transcriptionally inactive when the promoters of neighboring genes were removed in the cloning process. These genes are indicated in Table 3 as dashed arrows. B-type ARSs were also catalogued according to their local transcriptional activity. In Table 4, information concerning the unstable plasmids isolated is listed in the same way as it is found in Table 3. Here, 32/45 (71%) of B-type ARSs localize in transcriptionally active zones.

Combining the data from Table 3 and Table 4, it is apparent that 32/40 (80%) of ARSs localized in active transcription zones are B-type, whereas 16/29 (55%) of ARSs localized in inactive transcription zones are A-type (Table 5). These results suggest that transcriptional activity interferes with ARS activation, whereas transcriptional inactivity has little or no influence on ARS activation. In the latter situation, differential ARS activity must be determined by factors other than transcriptional activity.

**Table 5:**
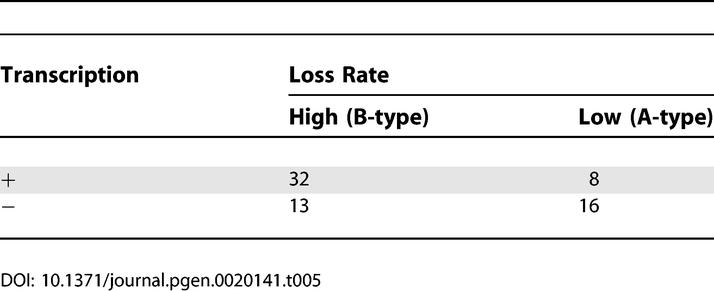
Categorization of ARSs Based on Plasmid Loss Rates and Transcriptional Activity

To evaluate the statistical significance of the correlation between flanking gene orientation and ARS activity, we used the two-sided Fisher exact formula to test the null hypothesis (i.e., there is no correlation between flanking gene transcription and ARS activity [30]). This analysis yielded a *p* value of 0.0018, indicating that the correlation between transcription towards a given ARS and the inability of that ARS to function in the *mcm1–1* mutant background is statistically significant.

A plausible explanation for this phenomenon is that a transcription bubble disrupts or prevents the assembly of the pre-RC as it traverses through the ARS [31]. In wild type cells, the pre-RC is able to reassemble efficiently at the ARS, and replication initiation is unaffected by the traversing transcription bubble. In contrast, in the *mcm1–1* mutant, pre-RC assembly at ARSs is already compromised [8]. Further interference by the traversing transcription bubble prevents or sufficiently delays the reassembly of the pre-RC. As a result, initiation occurs infrequently at those sites.

Correlation of the efficiency of initiation of ARSs with local transcription profiles provides an approximate summary of our data, but this correlation is not absolute. There are exceptions. For example, plasmids #40 and #101 contain ARS1009 and ARS1018, respectively (Table 3). In both cases, there is a gene oriented on one side of the ARS such that transcription of that gene is directed towards the ARS—a characteristic generally associated with B-type or “unstable” ARSs. However, each of these plasmids was isolated from a culture of *mcm1–1* cells that had been enriched for stable plasmids. Subsequent mcm assays confirmed that these plasmids are indeed stable in the mutant cells. When the neighboring genes were examined further, it was found that they both encode stress-response factors. Since these cells were grown under optimal laboratory conditions, it is likely that these genes were not being actively transcribed. Other exceptions may involve genes that are not transcribed during G_1_ phase when the pre-RC is assembled, or efficient transcription terminators protect ARS regions from transcription interference. Thus, gene orientation alone is not an accurate indicator of local transcriptional activity surrounding the replication origin.

### Obliteration of Transcription of Neighboring Genes of B-Type ARSs Confers Stability to Plasmids

Pre-RCs are assembled at replication origins during G_1_ phase [32]. Our hypothesis for the unsuccessful initiation of B-type ARSs in the *mcm1–1* mutant is that transcriptional activity neighboring those ARSs further cripples an already compromised pre-RC assembly process. As a result, assembly of the pre-RC at B-type ARSs becomes a limiting step in the activation of replication initiation during the G_1_–S phase transition.

If this hypothesis is true, two predictions can be made. First, reducing or obliterating the transcriptional activity surrounding a B-type ARS would increase the initiation efficiency of that ARS. Second, conditions that compromise the assembly of pre-RCs would suppress replication initiation at B-type ARSs but would have little or no effect on ARSs located in transcriptionally quiescent regions.

**Figure 4:**
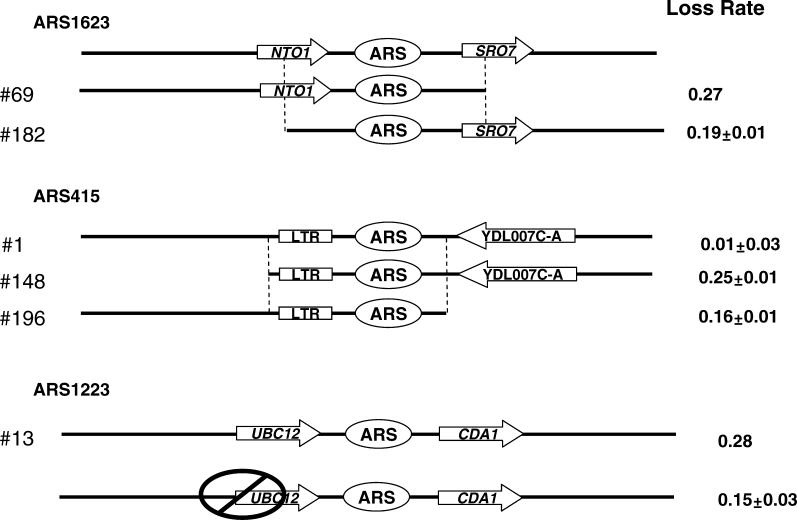
Effect of Local Transcription Pattern on Plasmid Stability Three sets of deletion plasmid constructs and their corresponding loss rates in *mcm1–1* cells are shown.

To test the first prediction, we took advantage of sets of plasmids containing a specific ARS that had been isolated independently on multiple plasmids but showed vastly different stabilities. As shown in Figure 4, two such plasmids containing ARS1623, #69 and #182, were isolated. This ARS lies in an intergenic sequence between two tandem genes where the *NTO1* gene is transcribed towards the ARS. As a result of the restriction digestion used to construct the library, plasmid #69 contains a full-length *NTO1* gene and promoter, while plasmid #182 is missing the promoter and the first 932 bp of the *NTO1* gene. As predicted by our hypothesis, truncation of the *NTO1* gene resulted in increased plasmid stability.

Another example can be found in a set of plasmids containing ARS415. ARS415 lies directly downstream of the *YDL007C-A* gene. Plasmid #148 contains both the ARS and the gene, while plasmid #196 carries only ARS415 without the upstream gene. Again, as predicted by our hypothesis, plasmid #196 shows a reduced plasmid loss rate in comparison to plasmid #148. Interestingly, plasmid #1, which contains a 11.4-kb insert, is stable even in the presence of the upstream promoter. This is a good example of the influence of insert size on plasmid stability (Figure 3A), although the underlying mechanism remains obscure.

Obviously, the plasmid-destabilizing sequences described in the previous two examples could be due to elements on the insert other than the promoter sequence directly adjacent to the ARS. To test our hypothesis without these possible artifacts, we strategically replaced the promoter region of a gene adjacent to a B-type ARS with a promoterless sequence. ARS1223 on plasmid #13 lies directly downstream of *UBC12* and has an elevated plasmid loss rate in *mcm1–1* cells (Figure 4). A portion (154 bp) of the promoter region of the *UBC12* gene that includes the first few base pairs of the coding region were replaced with a promoterless sequence of exactly the same length. This replacement resulted in a reduction in the plasmid loss rate in *mcm1–1*, suggesting that the *UBC12* promoter region is at least partially responsible for the inefficient initiation of ARS1223 in plasmid #13. It is important to point out that other factors are likely involved in modulating ARS activity because obliterating promoter function appears to have only a moderate effect on increasing plasmid stability. This notion is supported by the fact that the instability of about 30% of plasmids containing B-type ARSs cannot be accounted for by transcriptional interference (Tables 4 and 5).

### Preferential Usage of A-Type ARSs Is a Hallmark of Mutants Defective in the Assembly of the Pre-RC

**Figure 5:**
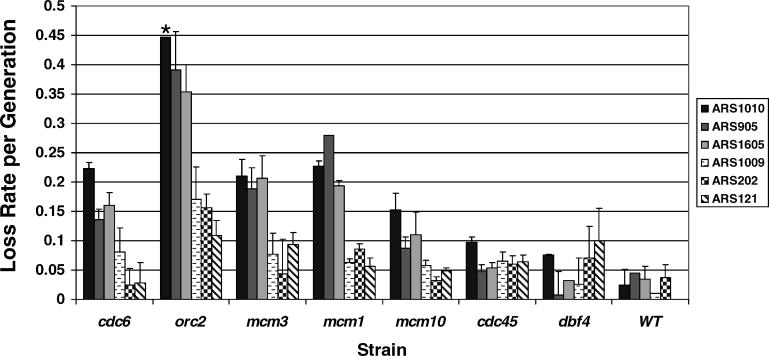
Comparing the Effect of *mcm1–1* on A-Type and B-Type ARSs with Those of Pre-RC Assembly *(cdc6, orc2,* and *mcm3)* and Activation *(cdc45* and *dbf4)* Mutants as Well as *mcm10* in a mcm Assay The asterisk indicates rate was too high to be measured. ARS1010, ARS905, and ARS1605 are B-type ARSs. ARS1009, ARS202, and ARS121 are A-type ARSs.

The second prediction of our hypothesis is that a compromise in the assembly of the pre-RC will result in the preferential usage of those ARSs devoid of transcriptional interference. Indeed, the effect of transcription in modulating the requirement of ARSs for pre-RCs had been reported in a recent study [31]. Initiation of DNA replication can be divided into two steps: assembly of the pre-RC during G_1_ phase, and activation of the pre-RC at the G_1_–S phase transition [32]. To test this hypothesis, we compared with *mcm1–1* the efficiency of replication of a set of A-type and B-type ARSs in mutants defective in the assembly of the pre-RC (*cdc6, orc2, mcm3)* [25,33] or in the activation of pre-RC *(cdc45, dbf4)* [34,35], respectively. We also included *mcm10* in this experiment. Mcm10 is believed to be recruited to replication origins after the Mcm2–Mcm7 helicase but before Cdc45 and Dbf4 [36–38]. If our hypothesis that assembly of the pre-RC is the limiting step of replication initiation at B-type ARSs, then only mutants defective in the assembly of the pre-RC should affect the initiation efficiency of these ARSs. Six plasmids containing A-type (ARS1009, ARS202, and ARS121) or B-type (ARS1010, ARS905, and ARS1605) ARSs were transformed into each of the mutant strains (Figure 5). Mutants defective in components of the pre-RC *(cdc6, orc2, mcm3)* show elevated plasmid loss rates for plasmids carrying ARS1010, ARS905, and ARS1605, but not for plasmids carrying ARS1009, ARS202, and ARS121, as observed for *mcm1–1.* While the severity of the defect varies from mutant to mutant, the set of ARSs tested show the same distinct hierarchy of efficiency of initiation among all of the pre-RC assembly mutants. Interestingly, the *mcm10* mutant shows an intermediate pattern, displaying detectable yet moderate differences between A-type and B-type ARSs. In contrast, this hierarchy of ARS usage is not observed in *cdc45* and *dbf4,* the pre-RC activation mutants. All six plasmids showed similar loss rates in these mutants (Figure 5). Furthermore, the loss rates (<0.1/cell division) are more comparable to those observed in wild-type than in the pre-RC assembly mutants. As predicted by our hypothesis, differentiation between A-type and B-type ARSs is only evident in the pre-RC assembly mutants but not in the pre-RC activation mutants. This finding suggests that the transcriptional interference observed in B-type ARSs is targeted only to the assembly process of pre-RCs. Once the pre-RC is activated, transcriptional interference is no longer in effect.

## Discussion

ARSs or replication origins do not initiate with equivalent efficiencies. This discrepancy in efficiency is enhanced in mutants that are defective in the early steps of DNA replication [5,6,25]. The molecular basis for this observation is unknown. Identifying the inherent determinants that define the initiation proficiency of an ARS requires the systematic characterization of a large number of ARSs that do (A-type) or do not (B-type) replicate efficiently in these mutants. Mcm1 has been shown to modulate the activity of replication origins directly by binding to replication origins [8,20] and indirectly by regulating the expression of multiple components of the pre-RC such as Cdc6 and Mcm7 [15,39]. By serial passages of a genomic library of ARSs through cultures of *mcm1–1* versus wild-type cells, 142 unique ARSs were cloned. Three of our clones represent new ARSs that have not been identified by previous genome-wide studies, suggesting that the current combined databases for ARSs [10] is about 98% complete.

Plasmids isolated from our genomic library indicate that without selective enrichment, about 20% of all ARSs belong to the A-type. However, this is likely an overestimate because removing transcriptional activity directed towards the ARS as a result of cloning may artificially stabilize 8/24 of the A-type ARSs that we isolated (Table 3). Based on this calculation, only 16/142, or about 11% of the ARSs from our screen may be bona fide A-type ARSs. This estimate is consistent with a previous genome-wide assessment that only 12% of all ARSs are located in intergenic regions between divergent promoters [10]. Assuming that there are approximately 350 replication origins per haploid yeast genome, we estimate that there may be as few as 40 A-type ARSs in the genome. Because *mcm1–1* shows no obvious growth defect (doubling time at 30 °C is 99 min compared to 96 min for wild-type cells), this result suggests that the B-type origins though firing inefficiently, are collectively making a significant contribution to the overall replication of the genome, and may explain the need for the large number of replication origins in yeast [40].

A major difference between A-type and B-type ARSs appears to be the polarity of transcription of adjacent genes with respect to the ARS. We found 67% of A-type plasmids contain ARSs located in intergenic regions devoid of transcriptional read through. Similarly, 71% of all unstable plasmids contain B-type ARSs located in intergenic regions 3′ of at least one of the adjacent genes. Indeed, simply inactivating the promoters of adjacent genes can enhance activity of B-type ARSs. This result suggests that transcription bubbles traversing through ARSs have a tendency to disrupt assembled pre-RCs. However, because reassembly of pre-RCs is a very efficient process, replication initiation is not affected in wild-type cells unless the assembly process is compromised as in the *mcm1–1* mutant, likely because of limiting amounts of Cdc6 and Mcm7 [15,39].

Individual examples supporting this genomic view have been well documented. Minichromosomes containing ARS1, a well-characterized B-type ARS that lies in the 3′ UTR of the *TRP1* gene, are unstable in the *mcm1–1* background [6,8]. A recent study showed that mutating the *TRP1* promoter stabilized plasmids containing ARS1 in *mcm2–1* cells [31]. In another study in which a galactose inducible promoter was placed upstream of the *TRP1* gene, the ability of ARS1 to initiate DNA synthesis was dramatically reduced in media containing galactose, but not in media containing glucose. Furthermore, insertion of a transcriptional terminator between the *TRP1* gene and ARS1 suppressed the transcriptional interference of *TRP1* [29]**.** Examples in human cells where active transcription inhibits DNA replication on a transfected plasmid have also been reported [41]. Similar results were found in Chinese hamster cells. A well characterized origin in that system lies in the intergenic region adjacent to the *DHFR* gene, which is normally transcribed during late G_1_–S phase of the cell cycle. Deletion of the DHFR promoter led to replication initiation from within the *DHFR* gene, a phenomenon not observed when this region is undergoing active transcription [42]. All three studies corroborate the idea that transcription through an origin reduces the replication initiation efficiency from that origin.

If inefficient initiation at B-type ARSs is only observed when assembly of the pre-RC is the limiting step in the initiation of DNA synthesis, then this phenomenon can be used to identify the stepwise assembly of gene products involved in the DNA replication initiation process. A panel of A-type and B-type ARSs were tested for their replication proficiency in a mcm assay using mutants defective either in the assembly of pre-RCs *(cdc6, orc2, mcm3)* or in the activation of pre-RCs *(cdc45, dbf4).* Indeed, only mutants defective in pre-RC assembly are able to differentiate A-type from B-type ARSs. Previous work showed that Mcm10 is recruited to replication origins after the recruitment of MCM2–7 but before the recruitment of Cdc45 and the Cdc7–Dbf4 kinase [37,38,43]. In this study, the *mcm10–1* mutant shows only a moderate ARS-specific minichromosome maintenance defect, consistent with the notion that Mcm10 is not directly involved in pre-RC assembly. However, our results suggest that Mcm10 may have a function in stabilizing the pre-RCs.

In the course of our analysis, we discovered that the overall size of a plasmid has a stabilizing effect on minichromosomes that may be independent of the content of the insert DNA sequence. Previous studies have shown that the size of both linear and circular minichromosomes affect their mitotic stabilities [44,45]. The best example is illustrated in the cloning of ARS1 on a large insert that stabilizes the minichromosome in the *mcm2–1* mutant [31] even though ARS1 in its native chromosomal location initiates poorly in the same genetic background [35]. Work in human cells has yielded similar results, where larger segments of DNA are replicated more efficiently [46].

Regulation of origin usage is an important aspect of the regulation of the rate of cell proliferation [2]. In our attempt to understand the underlying basis for the hierarchy of origin usage in *S. cerevisiae,* we found that the majority (~90%) of ARSs in the genome have attenuated usage when pre-RC assembly is compromised, and ~70% of these are due to transcriptional interference. Consistent with this observation, integrative computational analysis of genome-wide location studies [13] and cell cycle expression studies [47] indicate that pre-RCs tend to form in regions that are not actively transcribed during G_1_ phase [28]. G_1_-specific transcription may pose such severe interference with pre-RC assembly that initiation is all but eliminated at replication origins located in these regions, even under optimal conditions. The intimate relationship between transcription and replication initiation in the majority of replication origins ensures the coordination of these two processes in response to external stresses or nutrient availability. The corollary of this hypothesis is that in the complete absence of transcription, replication initiation may become promiscuous or unregulated as observed in amphibian embryos before the mid-blastula transition [48].

The findings in this study illustrate the complexity involved in the regulation of origin usage. Since this analysis used a plasmid-based approach, some of the complications encountered, namely the size discrimination, likely do not reflect the situation when the ARS is present at its normal chromosomal locus. Similarly, the correlation between gene orientation and replication efficiency does not take into account some of the details at each origin. For instance, strength of the neighboring promoters and timing of transcription during the cell cycle likely play a role in determining the ARS initiation frequency. It is also important to emphasize that transcriptional activity alone does not tell the whole story about the rules that govern the hierarchy of origin usage. The fact that ARSs located in transcriptional silent regions consist of both A and B types best illustrates this point (Table 5). Contribution by specific sequence elements may also be an important factor [8,20]. Further analyses combining motif search with DNA binding studies are needed to identify protein factors and DNA elements that determine the efficiency of replication initiation in *S. cerevisiae.*

## Materials and Methods

### Construction of genomic library.

The yeast genomic library used to screen for stable origins was constructed as follows. Yeast genomic DNA was prepared from W303 *mcm1–1* and W303 cells by centrifugation in CsCl as described [49]. Genomic DNA (4.5 μg) was digested with an empirically determined amount of DpnII (New England Biolabs, Beverly, Massachusetts, United States) at 37 °C for 4 min to yield DNA fragments with an average size of about 10 kb. DpnII cleaves GATC, a sequence that occurs at a probability of about 1 in 324 bp in the yeast genome. The enzyme was heat inactivated and the DNA was purified using a PCR Cleanup Kit (Qiagen, Valencia, California, United States). The DNA was then ligated into the unique BamHI site of pCL5 at room temperature for 30 min. The ligation mix was transformed directly into chemically competent Escherichia coli (Bioline, Little Clacton, United Kingdom) and left to grow overnight at 37 °C on six to eight LB + ampicillin plates. Typically, 300–500 colonies would grow on each plate. The colonies were then scraped into a pool of LB + ampicillin and grown for 4–6 h at 37 °C. Library plasmids were then extracted from the resulting culture using the Qiagen spin miniprep kit according to the manufacturer's protocol. This library construction was carried out eight times. In total, about 10,000 transformants with inserts were pooled in the analysis.

### Plasmid enrichments.

The appropriate genomic library plasmid mix was transformed into yeast cells and plated onto selective media and grown at 30 °C for 2 d. Liquid selective media was then spread on the plate to pool all of the transformants (typically 200–400 per plate). The pooled suspension of cells was used to inoculate a 5-mL culture of selective media. The culture was grown until saturated (usually 1–2 d at 30 °C). This was considered to be one enrichment, or passage. For additional enrichment, 20 μL of the saturated culture was used to inoculate a fresh 5-mL culture of selective media. Once saturated, this culture was considered to represent a second round of enrichment, or a second passage. This process was repeated up to as many as three passages.

### Screening for A-type or robust ARSs.

The yeast genomic library was transformed into 8534-M1 *mcm1–1* cells and plated onto selective media. The resulting colonies (typically 200–300) were pooled and passaged as described above. After two to three passages, plasmids were isolated from the culture according to the following protocol. Saturated yeast culture (1 mL) grown in selective media was spun down. The cell pellet was resuspended in 500 μL of Y1 buffer (Qiagen) supplemented with 250 μg zymolyase. This was incubated at 30 °C for 30 min, and then the resulting spheroplasts were spun down at 4,000*g* for 10 min. Plasmids were then extracted from the pellet using the Qiagen spin miniprep kit according to the manufacturer's protocol. The plasmids were eluted with 50 μL of water. A portion of the resulting plasmid mixture (5 μL) was transformed into chemically competent E. coli (Bioline) and plated on LB + ampicillin plates. The resulting colonies were grown overnight in LB + ampicillin media, and the plasmids were extracted using the Qiagen spin miniprep kit according to the manufacturer's protocol. The genomic insert present on each plasmid was sequenced using the following primers: PC5LlibseqF, 5′-gtcctgctcgcttcgctacttg-3′; and PC5LlibseqRnew, 5′-ccaccatacccacgccgaaacaa-3′. The resulting sequences were then aligned to the budding yeast genomic sequence available at http://www.yeastgenome.org to determine the exact length of the insert.

### mcm assays.

Yeast cells containing the appropriate plasmids were used to inoculate a 5-mL YPD culture. Before allowing the culture to grow, aliquots were plated on selective and nonselective media. The resulting colonies were counted and used to calculate the initial percentage of cells in the culture that have the plasmid (% I). The YPD culture was then incubated at 30° C for 24 h (usually about 13 generations). Aliquots of the culture were again plated on selective and nonselective media. The resulting colonies were counted and used to calculate the final percentage of cells in the culture that have the plasmid (% F). The number of generations *(n)* was calculated according to the following formula: *n* = log([I]/[F])/log(2), where [I] and [F] are the initial and final numbers of cells per milliliter in the culture, respectively. The loss rate (L) was calculated according to the formula: L = 1 − (% F / % I)^(1/n)^.

### Origin mapping.

The locations of origins found in the screen described in Table S1 were determined by comparison to published coordinates of known and predicted origins of replication [9,11,13] (C. Nieduszinski, unpublished data). Any region that contained an origin not listed in these studies was named according to its location with respect to other known origins.

### Promoter deletions.

The promoter of the *UBC12* gene was disrupted as previously described [50]. The resulting vector contains the promoterless *3-MYC* coding sequence in place of the endogenous promoter.

### Yeast strains.

For enrichment and cloning of A-type and B-type ARSs, the *mcm1–1* and isogenic wild-type strains of the 8543 genotype *(MATa bar1::URA3 ura3–52 leu2–3,-112 his4Δ34)* were used. Mutant strains *(cdc6–1, orc2–1, mcm3–10, mcm10–1, dbf4–1, cdc45–1)* and the isogenic wild-type strain used in Figure 5 have the W303 genotype *(ho ade2–1 trp1–1 can1–100 leu2–3,-112 his3–11,-15 ura3–52).*

## Supporting Information

Table S1 List of All ARS Inserts Analyzed Sites of colocalization of replication initiation proteins such as ORC and MCM proteins have been used as indicators of replication origins in a genome-wide study [13]. Systematic names were assigned to putative replication origins based on their positions along a particular chromosome [13]. Novel ARSs were given a systematic name to reflect their locations in the genome with respect to the locations of the published putative replication origins. Strain refers to strain from which plasmid was isolated. Cloned indicates ARS was subcloned or recloned by PCR for further analysis.
(53 KB XLS)

## Abbreviations

ARS: autonomously replicating sequence
mcm: minichromosome maintenance
pre-RC: prereplication complex
